# Continuous chest compression during sustained inflation versus continuous compression with asynchronized ventilation in an infantile porcine model of severe bradycardia

**DOI:** 10.1016/j.resplu.2024.100629

**Published:** 2024-04-09

**Authors:** Chelsea Morin, Tze-Fun Lee, Megan O'Reilly, Po-Yin Cheung, Georg M. Schmölzer

**Affiliations:** a Centre for the Studies of Asphyxia and Resuscitation, Neonatal Research Unit, Royal Alexandra Hospital, Edmonton, Alberta, Canada; b Department of Pediatrics, Faculty of Medicine and Dentistry, University of Alberta, Edmonton, Alberta, Canada

**Keywords:** Infant, Newborn, Pediatric, Chest Compression, Resuscitation, Sustained Inflation

## Abstract

**Background:**

Recently, the American Heart Association released a statement calling for research examining the appropriate age to transition from the neonatal to pediatric cardiopulmonary resuscitation approach to resuscitation.

**Aim:**

To compare neonatal and pediatric resuscitation approach by using either continuous chest compression with asynchronized ventilation (CCaV) or continuous chest compression superimposed with sustained inflation (CC + SI) during infant cardiopulmonary resuscitation. We hypothesized that CC + SI compared to CCaV would reduce time to return of spontaneous circulation (ROSC) in infantile piglets with asphyxia-induced bradycardic cardiac arrest.

**Methods:**

Twenty infantile piglets (5–10 days old) were anesthetized and asphyxiated by clamping the endotracheal tube. Piglets were randomized to CC + SI or CCaV for resuscitation (n = 10/group). Heart rate, arterial blood pressure, carotid blood flow, cerebral oxygenation, intrathoracic pressure and respiratory parameters were continuously recorded throughout the experiment.

**Main results:**

The median (IQR) time to ROSC with CC + SI compared to CCaV was 179 (104–447) vs 660 (189–660), p = 0.05. The number of piglets achieving ROSC with CC + SI and CCaV were 8/10 and 6/10, p = 0.628. Piglets resuscitated with CC + SI required less epinephrine compared to CCaV (p = 0.039). CC + SI increased the intrathoracic pressure throughout resuscitation (p = 0.025) and increased minute ventilation (p < 0.001), compared to CCaV. There was no difference in hemodynamic parameters between groups.

**Conclusions:**

CC + SI improves resuscitative efforts of infantile piglets by increasing the intrathoracic pressure and minute ventilation, and thus reducing the duration of resuscitation, compared to CCaV.

## Introduction

Cardiopulmonary resuscitation (CPR) occurs in approximately 1% of all neonatal and pediatric intensive care unit admissions and is associated with high morbidity and mortality.^1^, ^2^ Neonatal resuscitation guidelines recommend providing three chest compressions (CC) with a pause to deliver one inflation, resulting in a 3:1compression:ventilation ratio (3:1C:V).^3^, ^4^ This approach is tailored to the delivery room, in which respiratory failure is the most common cause of cardiac arrest.^3^, ^4^ Pediatric resuscitation guidelines recommend continuous CC with asynchronous ventilation (CCaV) after endotracheal intubation, which is tailored to focus on high quality CC, minimizing pauses in CC, while providing ventilation.^5^

Depending on the location of cardiac arrest (emergency room, neonatal or pediatric intensive care unit) in neonates (<28 days after birth) they are either resuscitated using the neonatal or pediatric resuscitation algorithm.^3^, ^5^ Neither the neonatal nor the pediatric resuscitation consensus on science and treatment recommendation provide information on when to use 3:1C:V or CCaV.^3^, ^5^ The approach used (3:1C:V or CCaV) varies based on location within the hospital (the neonatal intensive care unit most likely utilizes 3:1C:V, while the pediatric intensive care unit or emergency room would typically use CCaV), patient age, care provider expertise, or patient specific factors.^6^, ^7^, ^8^, ^9^, ^10^ Neonatal resuscitation guidelines suggest 3:1C:V may be used in the first 28 days of life, however 28 days is arbitrary and evidence-based age or timing to transition from 3:1C:V to CCaV is unknown.^6^ Several animal studies have compared 3:1C:V with CCaV for neonatal resuscitation and reported no difference in survival or time to return of spontaneous circulation (ROSC).^11^, ^12^, ^13^

For the last decade, we have studied a compression technique for neonatal and pediatric resuscitation using continuous CC with a constant high distending pressure, called a sustained inflation (CC + SI).^14^, ^15^, ^16^, ^17^, ^18^, ^19^, ^20^, ^21^ When CC + SI was compared to 3:1C:V in neonatal piglets, the time to ROSC and mortality was significantly reduced while regional and systemic hemodynamic and respiratory parameters were improved.^16^, ^22^, ^23^ Similarly, in two randomized trials in newborn infants receiving CPR in the delivery room, CC + SI reduced the time to ROSC compared to 3:1C:V.^19^, ^21^ Furthermore, CC + SI reduced time to ROSC and improved survival in pediatric piglets (aged 20–23 days, equivalent to 1 year old children) compared to CCaV.^14^, ^15^, ^24^ While these results are promising for the neonatal and pediatric patients, no study has examined this in infant piglets (equivalent to 28 days old infants). We aimed to compare outcomes CC + SI with CCaV during infantile CPR. We hypothesized that infantile piglets with asphyxia-induced bradycardic cardiac arrest resuscitated with CC + SI compared to CCaV would have shorter duration of resuscitation.

## Methods

**Fig. 1:**
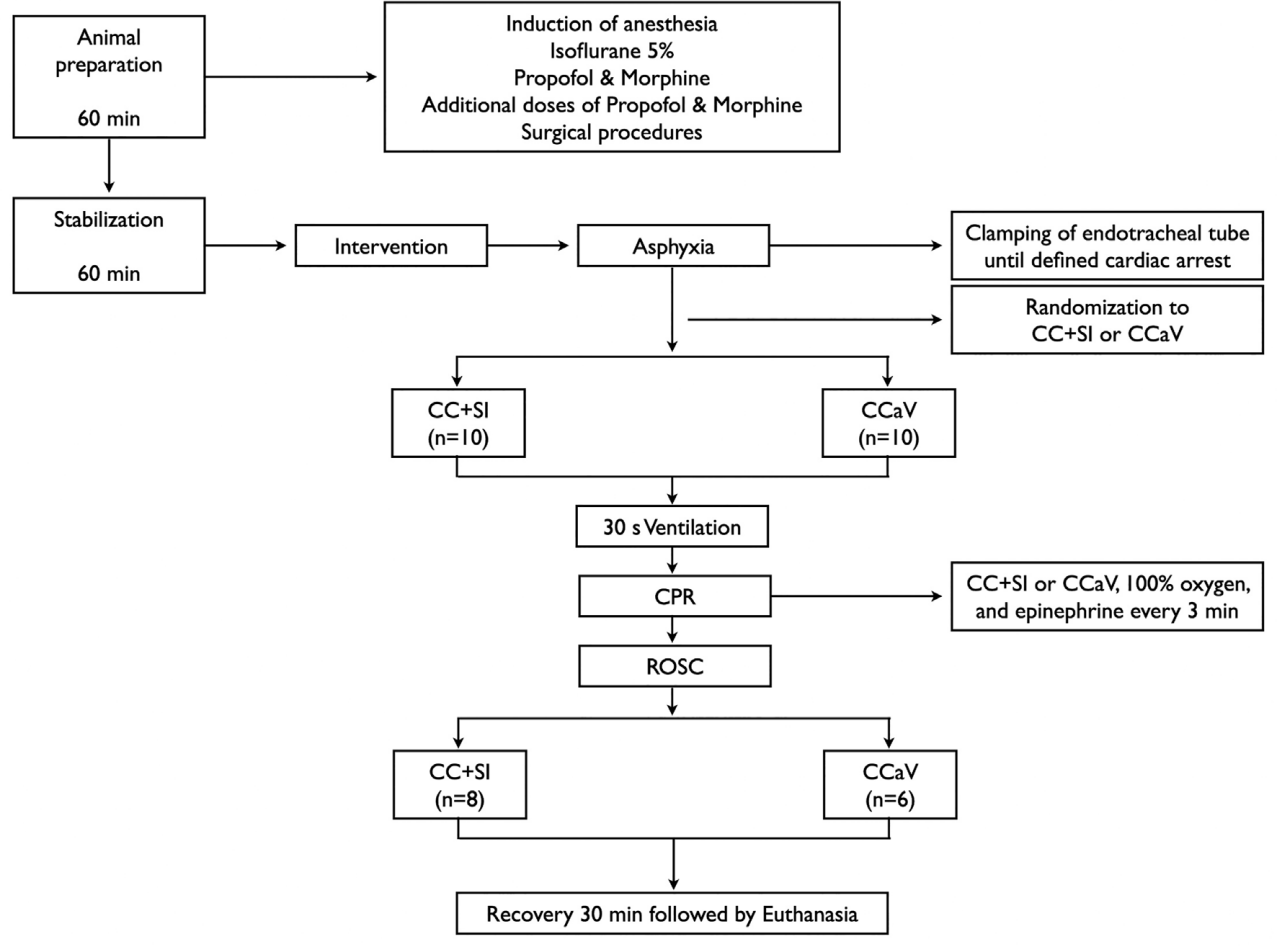
Study flow chart.

Twenty mixed breed pediatric piglets were obtained on the day of experimentation from the University Swine Research Technology Centre located in Edmonton, Alberta, Canada. All experiments were conducted in accordance with the guidelines and approval of the Animal Care and Use Committee (Health Sciences), University of Alberta (AUP4015), conducted and presented according to the ARRIVE guidelines,^25^ conducted according to the Canadian Council of Animal Care guidelines, and registered at preclinicaltrials.eu (PCTE0000479). A graphical display of the study protocol is presented in Fig. 1.

### Inclusion and exclusion criteria

Mixed breed pediatric piglets with a current age of 5–10 days old (weighing 2.9–4.1 kg), approximately equivalent to infants 25–35 days old, were included. We estimated 5–10 day piglets are approximately equivalent to infants 25–35 days based on our experience with their weight, size, and physiology. There was no exclusion criterion.

### Randomization

Piglets were randomly allocated to CC + SI or CCaV. Randomization was 1:1 using a computer-generated randomization program (https://www.randomizer.org). A numbered, sealed, brown envelope was opened just before the commencement of CPR containing the group allocation CC + SI or CCaV.

### Sample size and power estimates

Our primary outcome measure was the CPR time to achieve ROSC. Our pilot studies required a mean (SD) of 700 (95) seconds of CPR to achieve ROSC using CCaV. Based on this, a sample size of 20 piglets (10 per group) was sufficient to detect a clinically important (20%) reduction in time to achieve ROSC (i.e., 700 versus 560 seconds), with 90% power and a 2-tailed alpha error of 0.05.^26^

### Blinding

It was impossible to blind the team to the allocated intervention due to the nature of providing each intervention. However, the person (GMS) assessing bradycardic cardiac arrest was blinded to group allocation until after bradycardic cardiac arrest was confirmed. The statistical analysis was blinded to group allocation and only unblinded after the statistical analysis was completed.

### Animal preparation

Piglets were instrumented as previously described with modifications.^15^, ^16^, ^27^ Following the induction of anaesthesia using isoflurane, piglets were intubated via a tracheostomy, and pressure-controlled ventilation (Sechrist Infant Ventilator Model IV-100; Sechrist Industries, Anaheim, California) was commenced at a respiratory rate of 16–20 breaths/min and pressure of 20/5 cmH_2_O. Oxygen saturation was kept within 90–100%, glucose level and hydration was maintained with an intravenous infusion of 5% dextrose at 10 mL/kg/hr. During the experiment anaesthesia was maintained with intravenous propofol 5–10 mg/kg/hr and morphine 0.1 mg/kg/hr. Additional doses of propofol (1–2 mg/kg) and morphine (0.05–0.1 mg/kg) were also given as needed. The piglet’s body temperature was maintained at 38.5–39.5 °C using an overhead warmer and a heating pad.^15^, ^16^, ^27^

#### Intrathoracic pressure

An intrathoracic pressure probe was inserted in the intrapleural space just superior to the superior-most vertebral rib along the anterior axillary line to measure intrathoracic pressure. The intrathoracic pressure probe was secured with sutures.

### Hemodynamic parameters

A 5-French Argyle® (Klein-Baker Medical Inc. San Antonio, TX) double-lumen catheter was inserted via the right femoral vein for administration of fluids and medications. A 5-French Argyle® single-lumen catheter was inserted above the right renal artery via the femoral artery for continuous arterial blood pressure monitoring in addition to arterial blood gas measurements. The right common carotid artery was also exposed and encircled with a real-time ultrasonic flow probe (2 mm; Transonic Systems Inc., Ithica, NY) to measure cerebral blood flow.^15^, ^16^, ^27^

Piglets were placed in supine position and allowed to recover from surgical instrumentation until baseline hemodynamic measures were stable (minimum of one hour). Ventilator rate was adjusted to keep the partial arterial CO_2_ between 35–45 mmHg as determined by periodic arterial blood gas analysis. Mean systemic arterial pressure, systemic systolic arterial pressure, heart rate, and percutaneous oxygen saturation were continuously measured and recorded throughout the experiment with a Hewlett Packard 78833B monitor (Hewlett Packard Co., Palo Alto, CA).^15^, ^16^, ^27^

### Respiratory parameters

A respiratory function monitor (NM3, Respironics, Philips, Andover, MA) was used to continuously measure tidal volume (V_T_), airway pressures, gas flow, and end-tidal CO_2_ (ETCO_2_). The combined gas flow and ETCO_2_ sensor was placed between the endotracheal tube and the ventilation device. V_T_ was calculated by integrating the flow signal.^28^ ETCO_2_ was measured using non-dispersive infrared absorption technique. The accuracy for gas flow is ± 0.125 L/min, ETCO_2_ ± 2 mmHg.^29^

### Cerebral perfusion

Cerebral oxygenation (crSO_2_) was measured using the Invos^TM^ Cerebral/Somatic Oximeter Monitor (Invos 5100, Somanetics Corp., Troy, MI). The sensors were placed on the right forehead of the piglet and secured with wrap and tape. Light shielding was achieved with a slim cap. The Invos^TM^ Cerebral/Somatic Oximeter Monitor calculates crSO_2_, which is expressed as the percentage of oxygenated haemoglobin (oxygenated haemoglobin/total haemoglobin). Values of regional oxygen saturation are stored every second with a sample rate of 0.13 Hz.^30^, ^31^

### Experimental protocol

Piglets were randomized into two groups: “CC + SI” or “CCaV”. Asphyxia was induced by disconnecting the ventilator and clamping the endotracheal tube until bradycardic cardiac arrest was achieved, defined as mean arterial blood pressure < 20 mmHg and bradycardia (heart rate < 40% of baseline, extrapolated from neonatal and pediatric 2020 American Heart Association Guidelines for Cardiopulmonary Resuscitation and Emergency Cardiovascular Care of heart rate < 60, which is 40% of a baseline heart rate of ∼ 140).^14^, ^15^, ^24^ Once bradycardic cardiac arrest was confirmed, a numbered, sealed brown envelope containing the allocation “CC + SI” or “CCaV” was opened.

### Interventions

Thirty seconds after bradycardic cardiac arrest was diagnosed, positive pressure ventilation was performed for 30 sec using either a T-Piece (CC + SI group) or a self-inflating bag (CCaV) with 100% oxygen (as per group allocation), and after 30 sec of positive pressure ventilation, CC was started according to group allocation. CC was performed on a resuscitation board using the 2-thumb encircling chest compression technique.

During CC + SI (120/min compression rate), ventilations (sustained inflations) were provided for 30 sec each with a Neopuff T-Piece (Fisher & Paykel, Auckland, New Zealand), with default settings of peak inflating pressure of 30cmH_2_O, positive end expiratory pressure of 5cmH_2_O, and gas flow of 10 L/min. There was a 1 sec pause between successive inflations. Supplemental oxygen was 100% during CPR. During CCaV (120/min compression rate), asynchronous ventilations were delivered using a self-inflating bag (Laerdel, Stavanger, Norway), with 30 inflations per minute aiming to deliver a peak inflating pressure of 30cmH_2_O, measured with an attached manometer. CPR was continued for a maximum time of 11 min. The first dose of epinephrine (0.02 mg/kg per dose) was administered intravenously 2 min after the start of positive pressure ventilation. Subsequent doses of epinephrine (0.02 mg/kg) were given every 3 min until ROSC was observed. Bolus Ringer’s solution (3 mL) was given immediately after each dose of epinephrine.

ROSC was defined as an unassisted heart rate > 100/min for 15 sec, detected by femoral intra-arterial monitoring and ECG. After ROSC, piglets recovered and were monitored for 60 min while continuously ventilated. Blood gases were collected immediately after ROSC and after 60 min of recovery. At the end of experimentation (60 min), piglets were euthanized with an intravenous overdose of sodium pentobarbital (100 mg/kg).

### Data collection and analysis

Demographics of study piglets were recorded. Transonic flow probes, heart rate and pressure transducer outputs were digitized and recorded with LabChart® programming software (ADInstruments, Houston, TX). Airway pressures, gas flow, V_T_, and ETCO_2_ were measured and analyzed using Flow Tool Physiologic Waveform Viewer (Philips Healthcare, Wallingford, CT, USA).

The data are presented as mean (standard deviation) (SD) for normally distributed continuous variables and median (interquartile range - IQR) when the distribution was skewed. For all respiratory parameters, continuous values during CPR were analyzed. The data was tested for normality (Shapiro-Wilk and Kolmogorov-Smirnov test) and compared using Student’s *t-test* for parametric and Mann-Whitney *U*-test for nonparametric comparisons of continuous variables, and Fisher’s exact test for categorical variables. *P*-values are 2-sided and p < 0.05 was considered statistically significant. Statistical analyses were performed with SigmaPlot (Systat Software Inc, San Jose, USA).

## Results

Twenty newborn mixed breed piglets were obtained on the day of the experiment and were randomly assigned to CC + SI (n = 10) or CCaV (n = 10) group. Baseline characteristics are presented in Table 1.

**Table 1:** Baseline characteristics.

	SI (n = 10)	CCaV (n = 10)	p-value
Age (days)	8.0 (6.8–9.3)	8.0 (7–10)	0.299
Weight (kg)	3.6 (3.4–4.4)	3.3 (2.9–4.1)	0.089
Sex (male/female)	3/7	3/7	1.000
Heart rate (bpm)	206 (179–227)	210 (194–227)	0.385
MAP (mmHg)	72 (68–74)	76 (66–81)	0.508
Systolic pressure (mmHg)	98 (92–104)	106 (89–109)	0.120
Diastolic pressure (mmHg)	54 (50–60)	59 (49–62)	0.596
Carotid flow (mL/min)	75 (65–72)	72 (61–82)	0.078
Cerebral oxygenation (%)	40 (34–45)	40 (36–45)	0.743
pH	7.41 (7.38–7.44)	7.42 (7.41–7.46)	0.445
paCO_2_ (torr)	36 (35–38)	36 (33–37)	0.956
paO_2_ (torr)	74 (70–77)	70 (67–79)	0.754
Base excess (mmol/L)	−1.0 (-3.1 ∼ -0.6)	−0.7(-2.2 ∼ 0.5)	0.321
Lactate (mmol/L)	3.0 (2.2–4.5)	2.3 (1.9–3.5)	0.808

Data are presented as median (IQR); MAP – Mean arterial blood pressure, PaCO_2_ – partial pressure of arterial carbon dioxide, PaO_2_ – partial pressure of arterial oxygen.

### Resuscitation and primary outcome

Median (IQR) time to asphyxia from endotracheal tube occlusion was 331 (281–492)sec and 384 (237–439) with CC + SI and CCaV, respectively p = 0.850 (Table 3). At the end of asphyxia, all piglets had sinus bradycardia [median (IQR) heart rate of 74 (54–84) vs 75 (49–80) with CC + SI vs CCaV (p = 0.850)] and a mean arterial blood pressure of 20 (20–21) vs 19 (17–20) with CC + SI vs CCaV (p = 0.135).

**Fig. 2:**
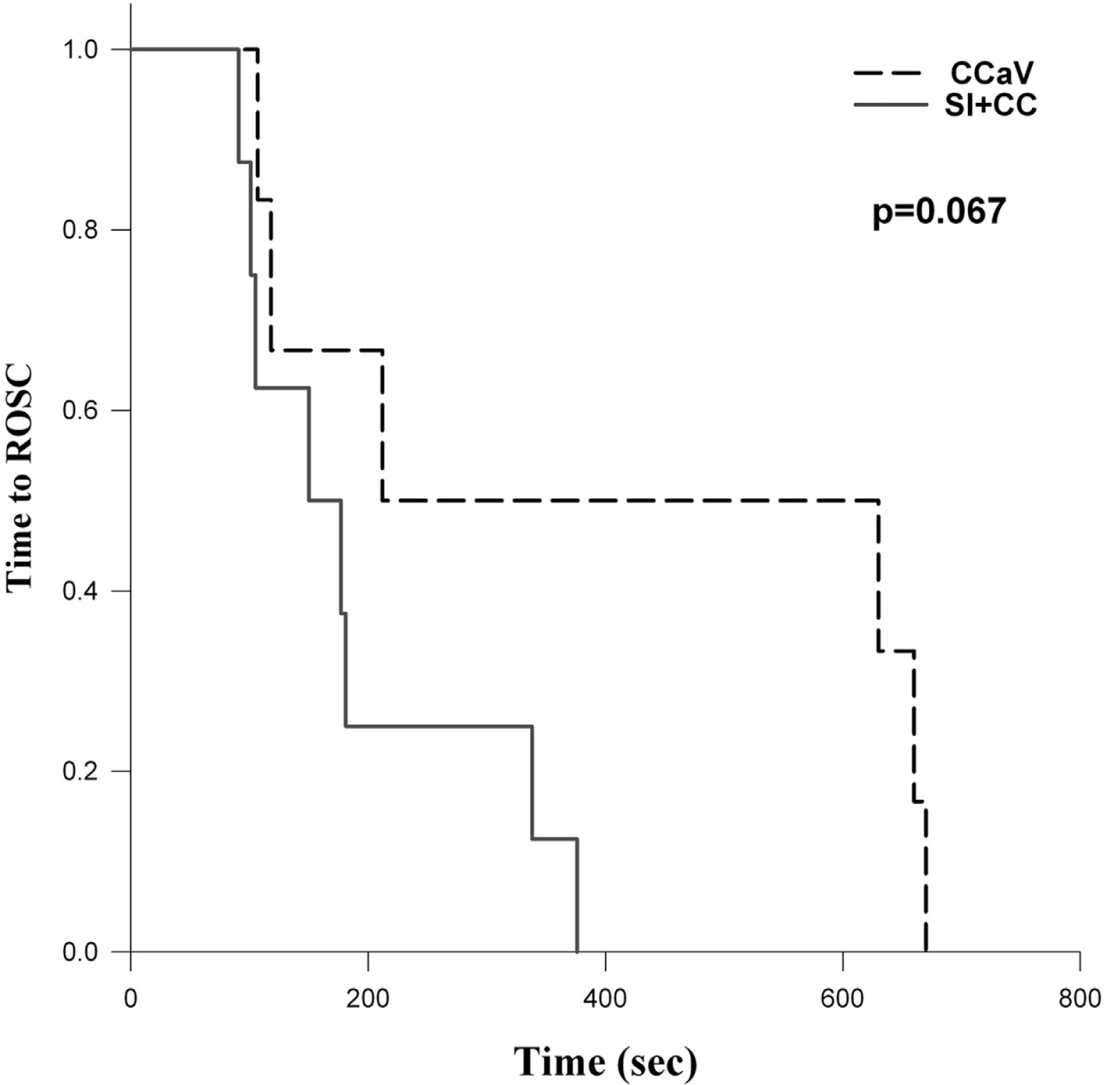
Time to ROSC Kaplan Meier Curve including only piglets achieving ROSC.

The rate of ROSC with CC + SI compared to CCaV was 8/10(80%) vs. 6/10(60%), respectively, p = 0.628 (Table 2). The median (IQR) duration of resuscitation with CC + SI compared to CCaV was 179 (104–447) vs 660 (189–660), p = 0.05 (Table 2). Fig. 2 represents a Kaplan–Meier curve depicting the duration of CC needed for animals achieving ROSC in both groups (p = 0.067).

**Table 2:** Characteristics of asphyxia and resuscitation of asphyxiated piglets.

		**CC + SI** **(n = 10)**	**CCaV** **(n = 10)**	**p value**
Asphyxia time (sec) ^†^		331 (281–492)	384 (237–439)	0.850
	Heart Rate (bpm)	74 (54–84)	75 (49–80)	0.850
	Mean arterial pressure (mmHg)	20 (20–21)	19 (17–20)	0.135
	pH^†^	6.97 (6.94–7.00)	6.96 (6.92–6.98)	0.365
	paCO_2_ (torr) ^†^	88 (86–91)	87 (79–97)	0.871
	Lactate (mmol/L) ^†^	16 (14–20)	15 (12–17)	0.159
	Base Excess (mmol/L) ^†^	−11 (-14 ∼ -10)	−12 (-15 ∼ -10)	0.266
Resuscitation	Requiring epinephrine	4 (40)	8 (80)	0.170
	Epinephrine doses ^†^	0 (0–2.3)	3 (0.8–3)	0.039
Achieving ROSC		8 (80)	6 (60)	0.628
ROSC time (sec) ^†^		179 (104–447)	660 (189–660)	0.05

Data are presented as n (%), unless indicated ^†^median (IQR).

**Fig. 3:**
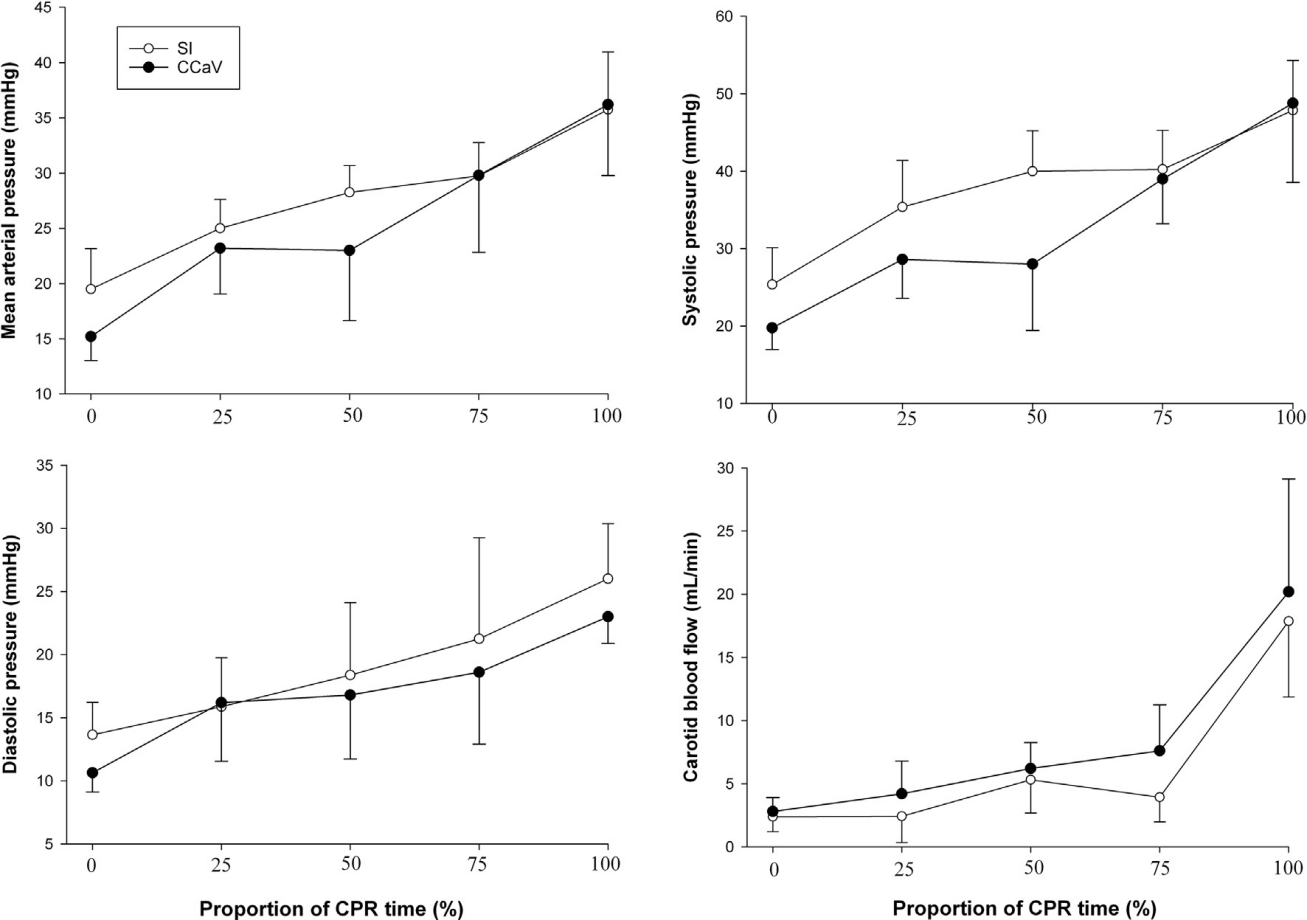
Hemodynamics parameters during CPR.

There was a significant difference in the number of epinephrine doses required for resuscitation, with CC + SI requiring fewer epinephrine doses compared to CCaV (0 (0–2.3) vs 3 (0.8–3), respectively, p = 0.039). Mean (SD) CC rate was 120 (3) and 120 (3) with CC + SI and CCaV (p < 0.001), respectively, and ventilation rate was 120 (3) and 30 (2) during CPR with CC + SI and CCaV(p < 0.001), respectively.

### Changes in hemodynamic parameters

**Fig. 4:**
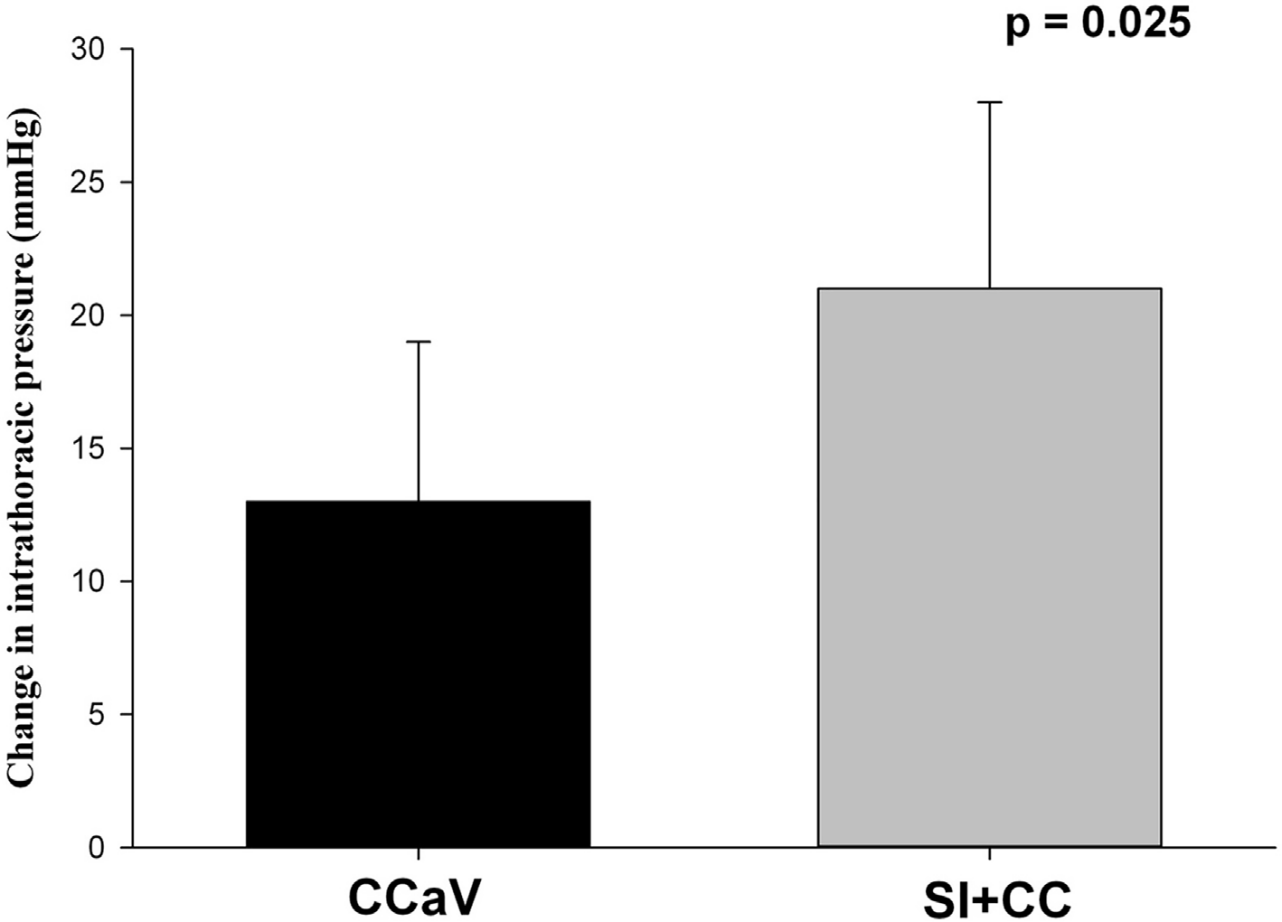
Maximum change in intrathoracic pressure (online supplement).

Hemodynamic parameters including heart rate, systolic, diastolic, and mean arterial blood pressure, carotid blood flow, and cerebral oxygenation were similar at baseline, after asphyxiation, during resuscitation, and during recovery between groups (Table 3 and Fig. 4). There was no difference in blood gas parameters at baseline, commencement of resuscitation, immediately after, or 60 min after ROSC (Table 4).

**Table 3:** Hemodynamic changes throughout the experiment.

	**SI + CC** **(n = 10)**	**CCaV(n = 10)**	**p-value**
**Baseline**			
Heart rate (bpm)	206 (179–227)	210 (194–227)	0.741
Mean arterial blood pressure (mmHg)	72 (68–74)	76 (66–81)	0.343
Systolic pressure (mmHg)	98 (92–104)	106 (89–109)	0.191
Diastolic pressure (mmHg)	54 (50–60)	59 (49–62)	0.596
Carotid flow (mL/min)	75 (65–84)	72 (61–82)	0.677
Cerebral oxygenation (%)	40 (34–45)	40 (36–45)	0.743
**At commencement of Resuscitation**			
Heart rate (bpm)	74 (54–84)	75 (49–80)	0.850
Mean arterial blood pressure (mmHg)	20 (17–23)	19 (17–20)	0.558
Systolic pressure (mmHg)	25 (23–32)	23 (22–26)	0.210
Diastolic pressure (mmHg)	13 (12–17)	13 (13–15)	0.569
Carotid flow (mL/min)	3 (3–5)	5 (3–5)	0.355
Cerebral oxygenation (%)	15 (15–15)	15 (15–15)	1.000
**Immediately after return of spontaneous circulation**			
Heart rate (bpm)	168 (140–174)	145 (101–179)	0.317
Mean arterial blood pressure (mmHg)	49 (32–52)	42 (26–52)	0.628
Systolic pressure (mmHg)	61 (44–65)	55 (38–62)	0.755
Diastolic pressure (mmHg)	35 (26–37)	29 (20–33)	0.268
Carotid flow (mL/min)	21 (19–28)	24 (22–29)	0.202
Cerebral oxygenation (%)	32 (30–37)	35 (32–40)	0.343
**60 min after return of spontaneous circulation**			
Heart rate (bpm)	268 (242–284)	303 (248–309)	0.415
Mean arterial blood pressure (mmHg)	69 (56–79)	68 (49–87)	0.371
Systolic pressure (mmHg)	89 (73–110)	90 (67–114)	1.000
Diastolic pressure (mmHg)	51 (34–63)	51 (38–74)	0.833
Carotid flow (mL/min)	52 (44–61)	39 (27–64)	0.149
Cerebral oxygenation (%)	34 (31–47)	37 (32–41)	0.927

Data are presented as median (IQR).

**Table 4:** Blood gas changes throughout the experiment.

	**CC_SI** **(n = 10)**	**CCaV(n = 10)**	**p-value**
**Baseline**			
pH	7.41 (7.38–7.44)	7.42 (7.41–7.46)	0.445
PaCO_2_ (torr)	36 (35–38)	36 (33–37)	0.956
PaO_2_ (torr)	74 (70–77)	70 (67–79)	0.754
Base excess (mmol/L)	−1.0 (-3.1 ∼ -0.6)	−0.7(-2.2 ∼ 0.5)	0.321
Lactate (mmol/L)	3.0 (2.2–4.5)	2.3 (1.9–3.5)	0.808
**At commencement of Resuscitation**			
Arterial pH	6.97 (6.94–7.00)	6.96 (6.92–6.98)	0.365
paCO_2_ (torr)	88 (86–91)	87 (79–97)	0.871
Base Excess (mmol/L)	−11 (-14 ∼ -10)	−12 (-15 ∼ -10)	0.266
Lactate (mmol/L)	16 (14–20)	15 (12–17)	0.159
**Immediately after return of spontaneous circulation**			
Arterial pH	6.94 (6.90–7.03)	6.97 (6.92–7.01)	0.705
paCO_2_ (torr)	61 (53–79)	60 (56–80)	0.701
Base Excess (mmol/L)	−17 (-19 ∼ -16)	−18 (-20 ∼ -13)	0.692
Lactate (mmol/L)	14 (13–16)	17 (13–20)	0.177
**60 min after return of spontaneous circulation**			
Arterial pH	7.32 (7.27–7.35)	7.26 (7.11–7.31)	0.567
paCO_2_ (torr)	41 (36–49)	48 (38–58)	0.625
Base Excess (mmol/L)	−6.3 (-7.9- −3.6)	−9.9 (-13 ∼ -2.9)	0.892
Lactate (mmol/L)	6.2 (4.8–7.9)	9.3 (4.1–13.3)	0.182

Data are presented as median (IQR).

**Fig. 5:**
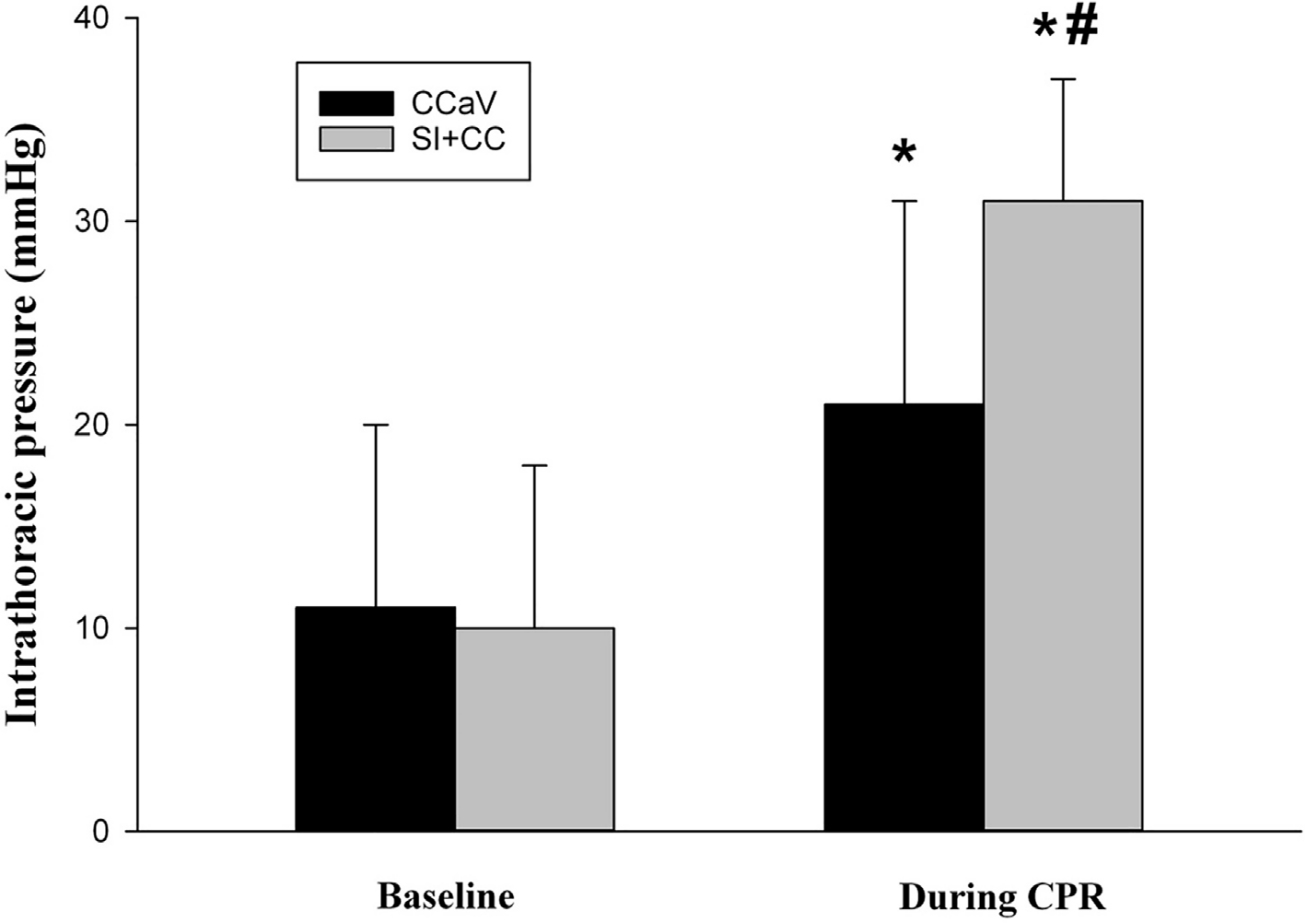
Mean (SD) maximum Intrathoracic pressure during CPR (online supplement).

Changes in intrathoracic pressure during CPR was significantly different in CC + SI compared to CCaV (Fig. 4, online supplement, p = 0.025), with CC + SI having a significantly higher intrathoracic pressure than CCaV (Fig. 5, online supplement, p < 0.05). Both groups had significantly higher (p < 0.05) intrathoracic pressures compared to their baseline values prior to CPR (Fig. 5, online supplement).

### Respiratory parameters

Respiratory parameters are presented in Table 5 (online supplement). In the CC + SI group, the mean (SD) V_T_ was 7.7 (1.9) mL/kg and in the CCaV group was 9.7 (2.6) mL/kg, p = 0.076. There was a significantly higher mean (SD) minute ventilation in the CC + SI group (697 (175)mL/kg/min vs 325 (70)mL/kg/min in CCaV, p < 0.001), which was secondary to a 4-fold increase in the number of inflations per minute (respiratory rate, p < 0.001, Table 5). Mean (SD) end-tidal CO_2_ and positive end-expiratory pressure were also significantly higher in the CC + SI group (47 (10)mmHg vs 25.5 (5)mmHg in CCaV, p < 0.001, and 36 (4)mmHg vs 0.5 (0.6)mmHg in CCaV, p < 0.001, respectively). In the CC + SI group mean (SD) peak inflation flow was significantly lower (10 (1)L/min in CC + SI vs 14 (1)L/min in CCaV, p < 0.001), however the peak inflation pressure was significantly higher (35 (4)mmHg in CC + SI vs 28 (6)mmHg in CCaV, p = 0.015).

**Table 5:** Respiratory parameter during cardiopulmonary resuscitation.

	**CC + SI** **(n = 10)**	**CCaV** **(n = 10)**	**p-value**
Peak Inflation Pressure (mmHg)	35 (4)	28 (6)	0.015
Positive End Expiratory Pressure (mmHg)	36 (4)	0.5 (0.6)	<0.001
Respiratory rate (/min)	120 (3)	30 (2)	<0.001
Peak Inflation Flow (L/min)	10 (1)	14 (1)	<0.001
Peak Expiratory Flow (L/min)	−16 (3)	−13 (1)	0.042
Tidal volume (mL/kg)	7.7 (1.9)	9.7 (2.6)	0.076
Minute ventilation (mL/kg/min)	697 (175)	325 (70)	<0.001
End-Tidal CO_2_ (mmHg)	47 (10)	25.5 (5)	<0.001

Data are presented as mean (SD).

## Discussion

While newborn infants requiring immediate resuscitative support in the delivery room are treated according to neonatal resuscitation protocols,^3^, ^4^ at some point after childbirth there will be a shift from the neonatal to the pediatric resuscitation protocols.^6^, ^10^ Owing to a lack of published recommendations guiding the transition from neonatal to pediatric resuscitation protocols, this transition time point of protocols is variable.^7^ This gap exists due to insufficient evidence indicating the ideal timing and circumstances for choosing between neonatal and pediatric resuscitation protocols.^6^, ^7^, ^8^, ^9^, ^10^ As a result, in the clinical setting, the responsibility for deciding which resuscitation protocols to follow lies with the healthcare team or the institution.^7^ Recently, the American Heart Association released a statement calling for research examining the appropriate age to transition from the neonatal to pediatric (CCaV) approach to resuscitation.^6^ While either 3:1C:V or CCaV might be used during infant CPR, neither technique has demonstrated reduced time to ROSC or improved survival in neonatal animal models.^11^, ^12^, ^13^

We have studied an alternative technique in which a high distending pressure (=sustained inflation) is applied continuously while providing CC, which results in faster time to ROSC, improved survival, and hemodynamic parameters, oxygen delivery, in pediatric animal models, while also allowing for passive ventilation.^14^, ^15^, ^24^ Furthermore, two randomized controlled trials have highlighted potential benefits of using CC + SI for newborn resuscitation in the delivery room.^19^, ^20^, ^21^ As such, in the current study we compared resuscitation with CC + SI to CCaV in an infantile aged piglet model of asphyxia-induced bradycardic arrest. The results of our study can be summarized as follows: i) survival rate with CC + SI vs CCaV was not different (Table 2, Fig. 2), however ii) CC + SI significantly reduced the time to achieve ROSC and reduced the amount of epinephrine required during resuscitation (Table 2; iii) hemodynamic and blood gas parameters were not different between CC + SI and CCaV throughout resuscitation (Table 3, Table 4, and Fig. 3); iv) CC + SI increased the intrathoracic pressure throughout resuscitation (Fig. 4, online supplement); and v) minute ventilation, and therefore alveolar oxygen delivery, was significantly increased with CC + SI (Table 5, online supplement). We speculate that increased minute ventilation, and therefore increased alveolar oxygen delivery, coupled with increased intrathoracic pressure may have contributed to the shorter duration of resuscitation in the CC + SI group.

To our knowledge, this is the first study comparing CC + SI and CCaV in the infantile aged piglet model. Several animal studies have compared CC + SI with CCaV during pediatric resuscitation, with all showing that CC + SI significantly improved survival and time to ROSC compared to CCaV.^14^, ^15^, ^24^ Additionally, several animal studies have demonstrated the beneficial effects of CC + SI in newborn resuscitation, when compared to the current recommended neonatal resuscitation protocol using 3:1C:V.^16^, ^17^, ^18^, ^22^ Furthermore, two randomized trials in newborn infants in the delivery room have also reported faster time to ROSC with CC + SI.^19^, ^20^, ^21^ In the current study using piglets approximately equivalent to human infants aged 25–35 days old, similar benefits of using CC + SI during resuscitation have been demonstrated: the duration of resuscitation was shorter [median duration of resuscitation (IQR): 179 (104–447) vs 660 (189–660) for CC + SI and CCaV, respectively p = 0.05], and in return the amount of epinephrine required was less [median number (IQR) of doses: 0 (0–2.3) vs 3 (0.8–3) for CC + SI and CCaV, respectively p = 0.039] (Table 3).

During CC + SI, a constant high distending pressure is applied during continuous CC, which results in continuous volume delivery, lung recruitment, and lung aeration.^22^, ^32^, ^33^ During CCaV there is less focus on providing adequate ventilation and the lung is deflated as each CC presses air out of the lung,^34^ resulting in lung de-recruitment. During chest recoil, there is a small amount of gas flow back into the lung. Asynchronous ventilations may cause decreased V_T_ delivery, result in atelectasis, and decrease or even reverse airflow in up to 65% of inflations.^35^ Indeed, in the current study approximately 59% of inflations during CCaV were compromised due to the interference between overlapping CC and ventilations. In a neonatal piglet model, Li *et al* reported a cumulated volume loss of 9.1 mL/kg occurred for each CCaV cycle.^22^ Furthermore, Li *et al* reported that V_T_ delivery was compromised in approximately 29% of inflations.^22^ During CC + SI, each CC will force V_T_ out of the lung, whilst during each chest recoil the air is drawn back into the lung.^15^, ^16^, ^22^ This resulted in a ventilation rate of 120/min (Table 5, online supplement), achieved passively through CC and recoil, which resulted in significantly higher minute ventilation and mostly likely contributed to the shorter duration of resuscitation.

The “thoracic pump theory” of the mechanics of CPR states that any maneuver which increases the intrathoracic pressure will result in blood flow during CPR.^36^ Raising the intrathoracic pressure can substantially improve carotid blood flow during CPR,^37^, ^38^ with the pressure gradient serving as the driving force for antegrade blood flow. Chandra *et al* showed that ventilation at high airway pressure (60–110 mmHg) while superimposing CC in an animal model increased carotid blood flow and arterial blood pressure.^37^, ^38^ Studies in preterm lambs demonstrated that a constant high distending pressure of 40cmH_2_O during ventilation increased intrathoracic pressure without impeding blood flow, and thus without compromising venous return.^39^ Similarly, we observed a higher carotid and pulmonary blood flow during CC + SI of asphyxiated newborn piglets with a distending pressure of 30cmH_2_O.^16^ In the current study we used a distending pressure of 30cmH_2_O during CC + SI resuscitation and observed a significantly higher intrathoracic pressure compared to CCaV (p = 0.025), although there were no significant differences in hemodynamic responses throughout CPR. However, the optimal distending pressure of approximately 25cmH_2_O for CC + SI has been established in neonates,^40^ this is the first study using CC + SI in infants, and as such, the optimal distending pressure in this population must still be studied.

### Limitations

All piglets were intubated, and CPR started 30sec after bradycardic cardiac arrest was confirmed to simulate a delay in recognition of the need for resuscitation and the start of resuscitation, which might not be possible in every setting. Additionally, although we blinded the bradycardic cardiac arrest assessor, we were unable to blind the intervention due to the difference in performing CC + SI and CCaV. We also did not assess neurologic outcomes in surviving piglets or collect tissue samples to assess for inflammation or damage.

## Conclusions

CC + SI improves resuscitative efforts of infantile piglets by increasing the intrathoracic pressure and minute ventilation, and thus reducing the time to achieve ROSC, compared to CCaV. This study provides support for further investigation into the use of CC + SI resuscitation in the neonatal-infantile aged time period. Further studies are warranted to assess infants born prematurely when > 44 weeks, and with underlying pathologies such as cardiac disease.

## CRediT authorship contribution statement

**Chelsea Morin:** Writing – original draft, Validation, Methodology, Investigation, Formal analysis, Data curation. **Tze-Fun Lee:** Writing – review & editing, Validation, Project administration, Methodology, Investigation, Formal analysis, Data curation, Conceptualization. **Megan O'Reilly:** Writing – review & editing, Validation, Resources, Project administration, Methodology, Investigation, Data curation, Conceptualization. **Po-Yin Cheung:** Writing – review & editing, Supervision, Resources, Methodology, Investigation, Formal analysis, Data curation, Conceptualization. **Georg M. Schmölzer:** Writing – review & editing, Validation, Supervision, Resources, Project administration, Methodology, Investigation, Formal analysis, Data curation, Conceptualization.

## Declaration of competing interest

The authors declare that they have no known competing financial interests or personal relationships that could have appeared to influence the work reported in this paper.
